# Robust Preimplantation Genetic Testing Strategy for Myotonic Dystrophy Type 1 by Bidirectional Triplet-Primed Polymerase Chain Reaction Combined With Multi-microsatellite Haplotyping Following Whole-Genome Amplification

**DOI:** 10.3389/fgene.2019.00589

**Published:** 2019-06-26

**Authors:** Mulias Lian, Caroline G. Lee, Samuel S. Chong

**Affiliations:** ^1^Department of Pediatrics, Khoo Teck Puat – National University Children’s Medical Institute, National University Health System, Singapore, Singapore; ^2^Department of Biochemistry, Yong Loo Lin School of Medicine, National University of Singapore, Singapore, Singapore; ^3^Cancer and Stem Cell Biology Program, Duke-NUS Graduate Medical School, Singapore, Singapore; ^4^Division of Medical Sciences, National Cancer Center, Singapore, Singapore; ^5^Department of Pediatrics, Yong Loo Lin School of Medicine, National University of Singapore, Singapore, Singapore; ^6^Department of Laboratory Medicine, National University Hospital, National University Health System, Singapore, Singapore

**Keywords:** preimplantation genetic testing, *DMPK*, myotonic dystrophy type 1, triplet-primed polymerase chain reaction, haplotype analysis

## Abstract

Myotonic dystrophy type 1 (DM1) is caused by expansion of the *DMPK* CTG trinucleotide repeat. Disease transmission to offspring can be avoided through prenatal diagnosis or preimplantation genetic testing for monogenic disorders (PGT-M). We describe a robust strategy for DM1 PGT-M that can be applied to virtually any at-risk couple. This strategy utilizes whole-genome amplification, followed by triplet-primed PCR (TP-PCR) detection of expanded *DMPK* alleles, in parallel with single-tube haplotype analysis of 12 closely linked and highly polymorphic microsatellite markers. Bidirectional TP-PCR and dodecaplex marker PCR assays were optimized and validated on whole-genome amplified single lymphoblasts isolated from DM1 reference cell lines, and tested on a simulated PGT-M case comprising a parent-offspring trio and three simulated embryos. Bidirectional *DMPK* TP-PCR reliably detects repeat expansions even in the presence of non-CTG interruptions at either end of the expanded allele. Misdiagnoses, diagnostic ambiguity, and couple-specific assay customization are further minimized by the use of multi-marker haplotyping, preventing the loss of potentially unaffected embryos for transfer.

## Introduction

Myotonic dystrophy type 1 (DM1; OMIM #160900) is the most common adult-onset muscular dystrophy with an overall estimated worldwide prevalence of 1 in 20,000, although much higher in regions with a founder effect such as Quebec (1 in 550) (Bird, 1999; Yotova et al., 2005). DM1 is caused by expansion of an unstable CTG trinucleotide repeat in the 3′ untranslated region of the *dystrophia myotonica protein kinase* (*DMPK*) gene located on chromosome 19q13.3 (Brook et al., 1992; Fu et al., 1992; Mahadevan et al., 1992). The disease is autosomal dominantly inherited, and presence of a full penetrance allele of 50 CTGs or above confirms the disease diagnosis (The International Myotonic Dystrophy Consortium, 2000). Repeat sizes smaller than 50 can be categorized into normal (5–34 CTGs) and mutable normal or premutation (35–49 CTGs) alleles. Premutation alleles are not known to cause disease but are prone to expand into the disease-causing full penetrance range in succeeding generations (Martorell et al., 2001).

DM1 is a multisystem disorder with highly variable clinical manifestations, spanning a continuum from mild (almost asymptomatic, cataracts, mild myotonia) to classic (muscle weakness, cataracts, myotonia, balding, cardiac arrhythmia) and congenital (infantile hypotonia, respiratory deficits, intellectual disability), as observed in individuals carrying 50–150 CTGs, 100–1,000 CTGs, and > 1,000 CTGs, respectively (Bird, 1999). DM1 individuals are also at an increased risk of several endocrine function abnormalities, including plasma testosterone insufficiency in males (Orngreen et al., 2012) and diminished ovarian reserve and poor response to ovarian stimulation in females (Srebnik et al., 2014; Fernandez et al., 2017). Due to the overlapping repeat sizes observed between phenotypic categories and lack of correlation between repeat size and age of onset beyond a size threshold (Savic et al., 2002), disease severity cannot be predicted solely on the basis of repeat size.

As congenital DM1 can be neonatally lethal and there is currently no treatment for DM1, transmission of this disorder can be avoided through pregnancy termination after prenatal diagnosis has determined inheritance of the expanded allele by the fetus. However, the decision to terminate an affected pregnancy is complicated by the fact that expansion size and disease severity are not tightly correlated. An alternative method to avoid disease transmission altogether is through preimplantation genetic testing for monogenic disorders (PGT-M). PGT-M involves direct or indirect testing for the disease-causing genetic mutation on samples biopsied from *in vitro*-fertilized day-3 (cleavage stage) or day-5 (blastocyst) embryos. Embryos diagnosed as unaffected with the disease can be transferred back to the mother’s uterus, such that any ensuing pregnancy will be unaffected. The most common strategy for DM1 PGT-M involves PCR amplification across the *DMPK* CTG repeat and selective transfer of embryos that do not carry the expanded allele for implantation (Sermon et al., 1997, 1998, 2001; Dean et al., 2001; Piyamongkol et al., 2001; Kakourou et al., 2007, 2008, 2010). However, because detection of the large expanded allele using regular repeat-spanning or flanking PCR is not possible, definitive diagnosis of an unaffected embryo relies on the detection of the affected parent’s non-expanded (i.e., normal) allele, which is only possible if it has a repeat size that is different from both alleles of the unaffected parent (Sermon et al., 1997, 1998; Dean et al., 2001; Piyamongkol et al., 2001; Kakourou et al., 2007, 2008). For couples with normal alleles of identical size, the triplet-primed PCR (TP-PCR) method first described by Warner et al. (Warner et al., 1996) has been utilized to detect the expanded *DMPK* allele directly in embryos (Sermon et al., 2001; Kakourou et al., 2008, 2010). Due to the risk of allele dropout (ADO), which is the random failure in amplification of one of the two *DMPK* alleles, up to four linked microsatellite markers (one upstream and three downstream) have been included in DM1 PGT-M cases, provided markers were informative (Dean et al., 2001; Piyamongkol et al., 2001; Kakourou et al., 2007, 2008, 2010). ADO of the expanded *DMPK* allele in particular can lead to diagnostic ambiguity or even misdiagnosis when TP-PCR is performed alone or when incomplete haplotype information is available. Analysis of microsatellite markers can also aid in the detection of parental or third-party DNA contamination which, if undetected, can lead to a misdiagnosis.

We propose a strategy that combines detection of the CTG repeat expansion by bidirectional TP-PCR with linked haplotype analysis generated from a dodecaplex marker panel (Lian et al., 2017), after whole-genome amplification (WGA). The aim of this strategy is to provide direct detection of the expansion mutation when present, while concurrently providing haplotype-based diagnostic confirmation, for virtually any DM1 PGT-M case. The use of multiple microsatellite markers also mitigates the risk of ambiguous haplotype phasing arising from insufficient informative markers.

## Materials and Methods

### Biological Samples

DM1 and non-DM1 cell lines were purchased from Coriell Cell Repositories (Coriell Institute for Medical Research, Camden, New Jersey, USA), and their genomic DNAs were extracted using the QIAsymphony DNA Midi Kit (Qiagen, Hilden, Germany) according to manufacturer’s instruction. The DM1 cell lines used were previously characterized DM1 reference materials which carry a mosaic small CTG repeat expansion (GM06075; 12, 56, 70 ± 0.9 repeats) or a large expansion (GM04648; 5, 1,008 ± 49 repeats, GM04567; 21, 637 ± 33 repeats, and GM03989; 13, 2000 repeats) (Kalman et al., 2013; Lian et al., 2015), whereas the non-DM1 cell lines used included GM16243, which carries GAA repeat expansions in the *frataxin* gene and was previously determined to carry 13 and 14 *DMPK*-CTG repeats (Lian et al., 2015), GM06890, GM06892, GM06852, GM09197, GM03813, GM03814, GM03815, AG17487, and GM00143, all of which are heterozygous for various normal-sized *DMPK* CTG repeat alleles. Isolation, lysis, and whole-genome amplification (WGA) of single cells from GM06075 (*n* = 21), GM04648 (*n* = 15), GM16243 (*n* = 5), GM06890 (*n* = 6), GM06892 (*n* = 4), GM06852 (*n* = 6), GM09197 (*n* = 4), GM03813 (*n* = 3), GM03814 (*n* = 3), GM03815 (*n* = 8), AG17487 (*n* = 4), and GM00143 (*n* = 6), as well as groups of six cells from GM06075 (*n* = 3) and GM04648 (*n* = 3), were performed as described (Lian et al., 2017). Aliquots of single-cell WGA products were used to validate bidirectional *DMPK* TP-PCR and dodecaplex marker panel PCR assays for direct CTG repeat expansion detection and linked multi-marker haplotype analysis, respectively. This study was approved by the Institutional Review Board of the National University of Singapore (Ref: 07-123E).

### Polymerase Chain Reaction Amplification

TP-PCR reactions were performed in 50-μl volumes, each containing 2 U HotStarTaq DNA polymerase (Qiagen), 2× Q Solution (Qiagen), 1× supplied PCR buffer (Qiagen), 0.2 mmol/L each of deoxynucleotide triphosphates (Roche, Penzberg, Germany), and either 100 ng of genomic DNA or 2 μl of WGA product as template. The 5′ TP-PCR reaction included 0.6 μmol/L each of primers *Fam*-P2 and P3R (Warner et al., 1996), and 0.06 μmol/L of primer 5′ TPR (Lian et al., 2015), whereas the 3′ TP-PCR reaction utilized 0.6 μmol/L each of primers *Fam*-3′ R and 3′ Tail, and 0.06 μmol/L of primer 3′ TPF (Lian et al., 2015). Thermal cycling was performed on the GeneAmp PCR System 9700 (Applied Biosystems-Life Technologies, Carlsbad, CA, USA) and included an initial enzyme activation at 95°C for 15 min, followed by 30 cycles of 98°C for 45 s, 60°C for 1 min, and 72°C for 5 min, with a final extension at 72°C for 10 min. The previously reported method for PGT-M of DM1 (Kakourou et al., 2010) was performed as described.

For TP-PCR analysis, a 2-μl aliquot of *Fam*-labeled product was mixed with 1 μl of GeneScan 500 ROX (Applied Biosystems) or MapMarker 1000 ROX (Bioventures, Murfreesboro, TN, USA) internal size standard and 8 μL of Hi-Di Formamide (Applied Biosystems), denatured at 95°C for 5 min, rapidly cooled to 4°C, and subjected to capillary electrophoresis (36 cm, POP-7, 5 s at 1 or 10 kV injection, 15 kV run) on the 3130xl Genetic Analyzer (Applied Biosystems). GeneScan electropherograms were analyzed using GeneMapper version 4.0 software (Applied Biosystems).

Dodecaplex marker panel genotyping was performed as previously described (Lian et al., 2017), except that the reaction was performed in a 50-μl volume containing either 10 ng of genomic DNA or 2 μl of WGA product.

### Simulated PGT-M Case

Archived genomic DNAs and multi-cell samples mimicking trophectoderm biopsies were obtained from the UK NEQAS (United Kingdom National External Quality Assessment Service) for Molecular Genetics 2014-15 simulated PGT-M proficiency test cycle. Each simulated trophectoderm biopsy sample, comprising six single lymphocyte cells, was lysed and whole-genome amplified as above. The simulated PGT-M case consisted of a DM1-affected male, his wife, an affected daughter, as well as simulated trophectoderm biopsies from three “embryos.” The parent-offspring trio’s genomic DNAs and simulated trophectoderm samples were analyzed using the validated 5′ and 3′ *DMPK* TP-PCR and dodecaplex marker PCR assays.

### Data Interpretation

**Figure 1:**
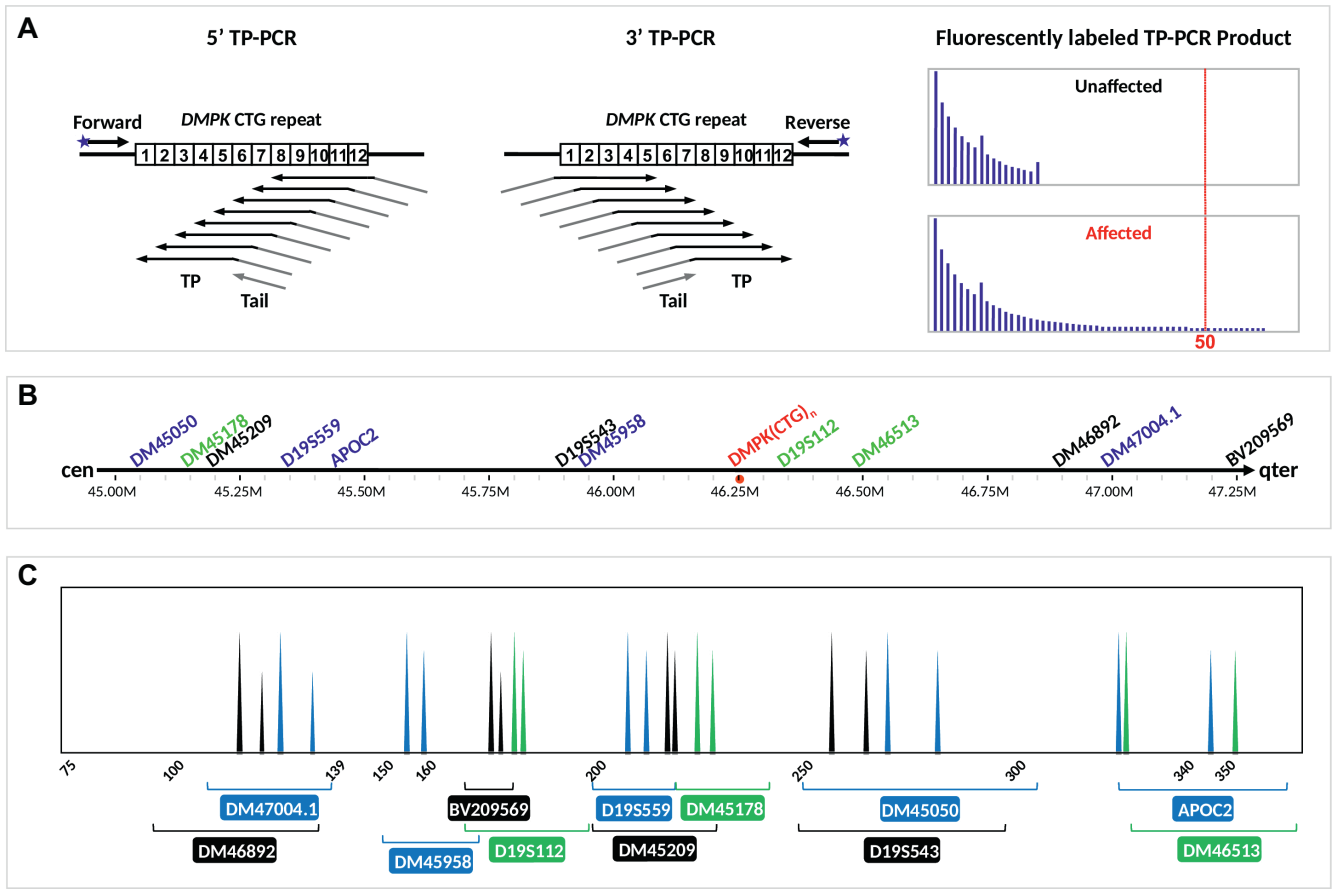
Schematic illustration of the DM1 PGT-M strategy. **(A)** Bidirectional 5′ and 3′ TP-PCR at the *DMPK* CTG repeat locus, depicting annealing positions of primers (arrows), as well as the expected electropherogram patterns from unaffected and DM1-affected embryos after capillary electrophoresis. **(B)** Location of 12 microsatellite markers relative to the *DMPK* CTG repeat. **(C)** Expected electropherogram results after multiplex PCR amplification of the dodecaplex marker panel and capillary electrophoresis, showing the electrophoretic peak color and approximate size range of each marker. Adapted from Lian et al. (2016, 2017).

TP-PCR uses three primers, i.e., a locus-specific flanking primer, a triplet-priming (TP) primer, and a non-specific tail primer. Due to random annealing of the TP primer within the CTG repeat, TP-PCR produces a series of products that differ from each other by 3-bp intervals (Figure 1A). The first capillary electrophoretic peak represents a 5-repeat amplified product as the TP primer anneals to the five repeats located nearest to the flanking primer, and each succeeding peak represents amplified product containing an additional triplet repeat. The TP primer consists of unique nucleotides at the junction between the five repeats and tail primer sequence that allow specific and strong annealing of the TP primer to its last possible annealing site within the repeat tract. The stronger annealing in turn allows more products to be amplified from the last annealing position, giving the last peak a taller peak height and highly accurate sizing of normal alleles and modest expansions. The repeat size of an allele can be easily derived by counting the clearly observable fluorescent peaks that are present. A result showing <50 repeats on the electropherogram indicates an unaffected embryo, whereas a result showing ≥50 repeats indicates a DM1-affected embryo.

The positions and expected PCR product peaks of the 12 flanking microsatellite markers relative to the *DMPK* CTG repeat are shown in Figures 1B,C, respectively. Haplotype phasing was performed by assigning the paternally derived allele of each marker’s genotype that is observed in the DM1-affected daughter as the affected allele. The series of 12 paternal alleles in the affected daughter was designated as the mutant/disease haplotype.

## Results

### Validation of Bidirectional *DMPK* TP-PCR and Dodecaplex Marker PCR Assays on Whole-Genome Amplified Single Lymphoblasts

**Figure 2:**
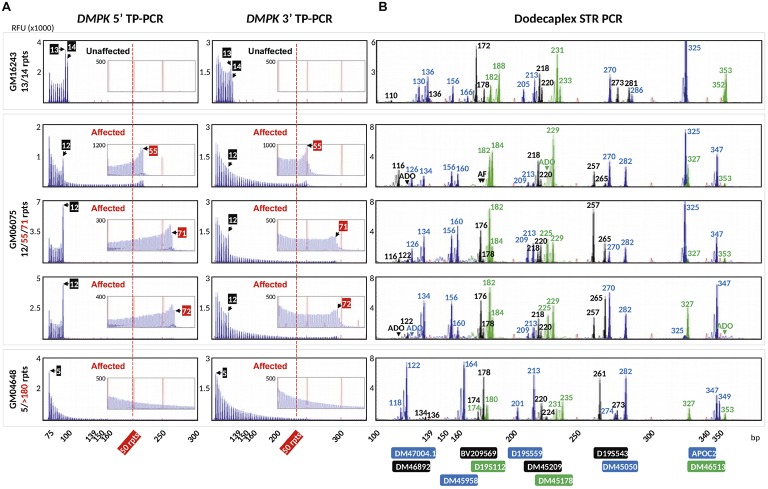
Electropherograms of whole-genome amplified single lymphoblasts from three cell lines after bidirectional 5′ and 3′ *DMPK* TP-PCR **(A)**, and dodecaplex marker panel PCR **(B)** amplifications. A non-DM1 cell line (top row), a DM1-affected cell line carrying a modest expansion (rows 2–4), and a DM1-affected cell line carrying a large expansion (row 5) were used. The 50-repeat cutoff for full penetrance *DMPK* allele is represented by a vertical dotted red line. Numbers in the TP-PCR and dodecaplex marker panel PCR electropherograms indicate CTG repeat number and product fragment size in base pairs, respectively. RFU, relative fluorescence units; ADO, allele dropout; AF, amplification failure.

**Figure 3:**
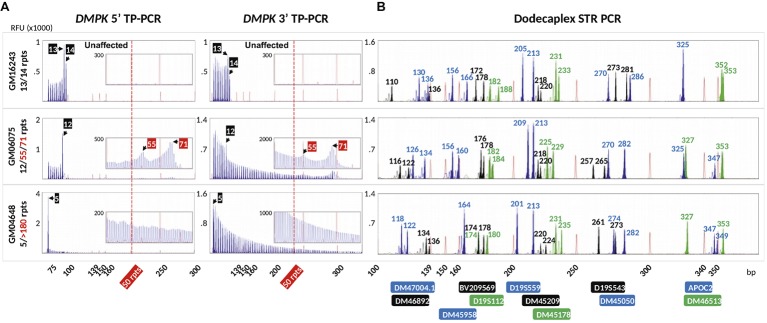
Electropherograms of cell line-extracted genomic DNA after bidirectional 5′ and 3′ *DMPK* TP-PCR **(A)** and dodecaplex marker panel PCR **(B)** amplifications. RFU, relative fluorescence units.

**Figure 4:**
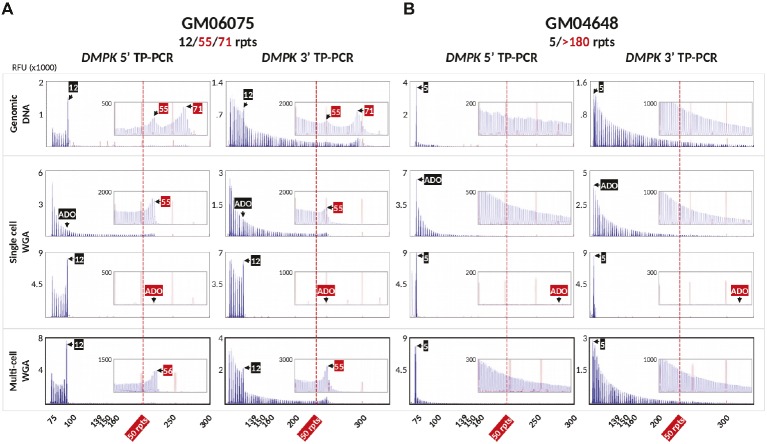
Bidirectional 5′ and 3′ *DMPK* TP-PCR electropherograms of GM06075 **(A)** and GM04648 **(B)** genomic DNAs (row 1; also shown in Figure 3), whole-genome amplified single lymphoblasts showing allele dropout of the normal allele (row 2), allele dropout of the expanded allele (row 3), and whole-genome amplified multi-cell samples with no allele dropout (row 4). RFU, relative fluorescence units; ADO, allele dropout.

To validate the DM1 PGT-M strategy of combined TP-PCR and dodecaplex marker PCR assays, a total of 41 single lymphoblasts from non-DM1 and DM1 cell lines were subjected to whole-genome amplification (WGA), and aliquots of WGA product were used to perform the three assays in parallel. Both 5′ and 3′ *DMPK* TP-PCRs confirmed the presence of two normal alleles and absence of an expansion in non-DM1 cells, and presence of one normal allele and one expanded allele of >50 CTGs in cells with small or large expansions (Figure 2A). The results from single-cell WGA products were consistent with those obtained from cell line-derived genomic DNA (Figure 3), thus indicating that both TP-PCR assays can be used with confidence on single-cell WGA products. WGA products from an additional 44 heterozygous single-cells were included for the purpose of calculation of amplification efficiency and ADO rate of the *DMPK* allele. Amplification efficiency at the *DMPK* CTG repeat was 100%, while ADO of either the normal or expanded allele was observed in 19 of 85 cells (22.4%) (Figure 4). The assays were also performed on six multi-cell samples, all of which amplified successfully without ADO (Figure 4).

Dodecaplex marker genotyping of the single-cell WGA products revealed that GM06075 was heterozygous for all markers, whereas GM16243 and GM04648 were heterozygous for 11 markers (Figure 2B). The ADO rates of the markers ranged from 2.4 to 26.8%.

**Figure 5:**
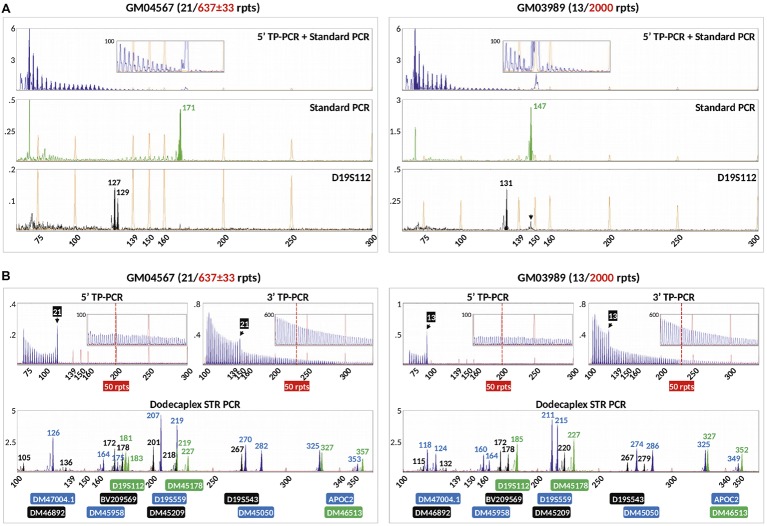
Comparison of the modified TP-PCR and linked marker assay of Kakourou et al. (2010) **(A)** and our combined bidirectional TP-PCR and linked multi-marker assay **(B)** on two DM1-affected samples. The modified TP-PCR consisted of 5′ TP-PCR amplification of *DMPK* locus using P2/P4CAG/P3R primers (top lane), standard PCR amplification of *DMPK* locus using P2/DMPK2 primers (middle lane), and PCR of linked marker D19S112 (bottom lane). Black arrow indicates bleed-through signal from VIC channel that was initially misidentified as a second D19S112 allele in GM03989.

We compared our method with a previously reported modified TP-PCR (mTP-PCR) protocol for PGT-M of DM1 (Kakourou et al., 2010) using genomic DNA from GM04567 and GM03989, which are both known to carry a normal and a large expanded *DMPK* allele. This protocol, which simultaneously amplifies the *DMPK* CTG repeat and a linked marker (D19S112), produced a diminishing TP-PCR ladder pattern from the 5′ TP-PCR, although the normal alleles could not be clearly identified from the continuous peak ladder, which also did not extend beyond 50 repeats (Figure 5A). The standard PCR detected only normal allele fragments of 171 and 147 bp from GM04567 and GM03989, respectively. PCR amplification of D19S112 showed that GM04567 was heterozygous as it produced 127- and 129-bp fragments, while GM03989 was homozygous for a 131-bp fragment. Another larger fragment of ~147 bp detected in GM03989 (Figure 5A, black arrow) was determined not to be a second allele of the marker, but was caused by bleed-through from the 147-bp VIC-labeled standard PCR product of the normal *DMPK* CTG repeat allele. The homozygosity of GM03989 for the marker was confirmed by simplex PCR (data not shown) as well as by our dodecaplex marker panel (Figure 5B).

In marked contrast, our 5′ and 3′ TP-PCR profiles of GM04567 and GM03989 clearly showed the 21- and 13-repeat normal alleles, respectively, while the peak ladders extended well beyond 50 repeats, unambiguously indicating the presence of full mutation expansions (Figure 5B). Furthermore, our dodecaplex marker panel was heterozygous at ten and nine loci in GM04567 and GM03989, respectively.

### Simulated PGT-M

**Figure 6:**
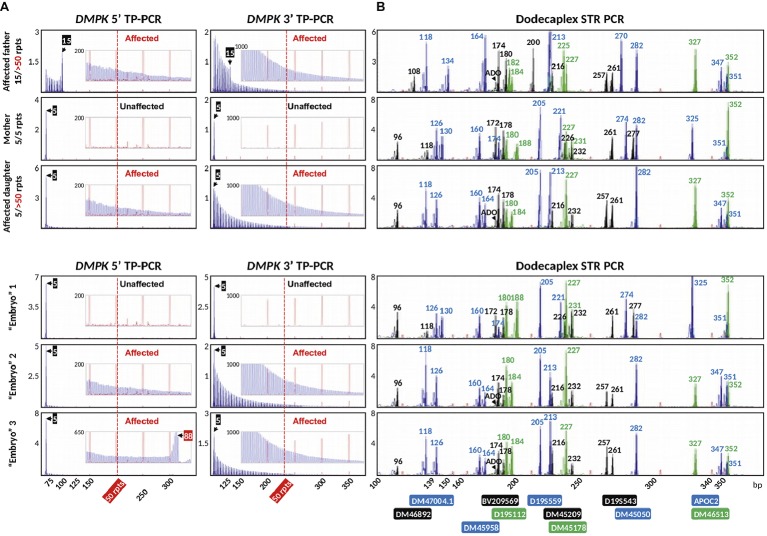
Bidirectional *DMPK* TP-PCR **(A)** and dodecaplex marker panel PCR electropherogram **(B)** profiles from a UKNEQAS simulated PGT-M case, which consisted of a parent-child trio and three simulated trophectoderm samples. ADO, allele dropout.

We applied the validated bidirectional *DMPK* TP-PCR assays and the dodecaplex marker panel assay to the archived genomic DNA samples from a previous UK NEQAS proficiency test, consisting of a parent-offspring trio (affected father, unaffected mother, and an affected daughter), and the archived WGA product from three simulated trophectoderm samples each consisting of six lymphoblasts. Both 5′ and 3′ *DMPK* TP-PCRs confirmed the presence of an expanded allele of >50 repeats in the DM1-affected father and daughter, while the mother was expansion-negative (Figure 6A). The repeat size of the father’s normal allele was found to be 15 CTGs, whereas the mother was homozygous for the 5-repeat normal alleles. The daughter inherited the paternal expanded allele and maternal 5-repeat normal allele.

**Figure 7:**
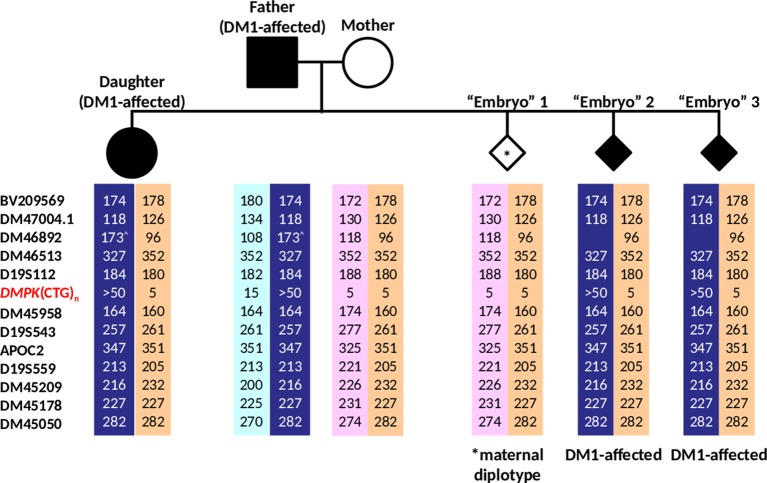
Haplotypes generated from analysis of 12 linked markers in the simulated PGT-M case. Haplotype pairs of the DM1-affected father, unaffected mother, affected daughter, and three simulated “Embryo” samples are shown. Markers are ordered from the telomere (top) to centromere (bottom) of the q-arm. Dark blue- and light blue-shaded columns denote haplotypes linked to the paternal mutant/expanded and normal alleles, respectively. Light purple- and orange-shaded columns denote haplotypes linked to both of the maternal normal alleles. ^^^Allele size determined from simplex PCR.

The dodecaplex marker PCR assay successfully amplified all 12 linked markers from the DNAs of the parent-daughter trio (Figure 6B). ADO of the paternal 173-bp DM46892 allele was however observed in both the affected father and affected daughter, most likely a result of poorer amplification yield compared to the much smaller other allele in father (108 bp) and daughter (96 bp) and overlapping stutter peaks of the next marker with the same fluorophore tag. Four upstream markers (BV209569, DM47004.1, DM46892, D19S112) and one downstream marker (DM45209) were fully informative, i.e., all four parental alleles are dissimilar. Another upstream marker (DM46513) and four downstream markers (D19S543, APOC2, DM45178, DM45050) were semi-informative, i.e., one allele is shared between father and mother (Figures 6B, 7). The remaining two markers (DM45948 and D19S559) were not informative.

Of the three simulated embryo trophectoderm samples, TP-PCR results for embryos 2 and 3 showed electrophoretic peaks that extended beyond 50 repeats, indicating that they were “expansion-positive” and thus affected (Figure 6A). It was also noted that the peaks in the 5′ TP-PCR result of embryo 3 were interrupted by a gap of ~5 peaks after 88 CTGs, indicating a possible interruption near the 5′ end of the expanded allele’s repeat. An expanded allele was not detected in embryo 1, which showed only the maternal 5-repeat peak. This result was suggestive of either allele dropout (ADO) of the paternal allele or maternal-only genetic contribution, and hence a definitive diagnosis was not possible from the TP-PCR results.

The dodecaplex marker PCR results of the three simulated embryo trophectoderm samples are presented in Figure 6B. As the affected daughter was available to establish the parental diplotypes, the marker haplotype linked to the paternal expanded allele could be ascertained (Figure 7). With five fully informative and five semi-informative markers, it was possible to deduce that the paternal haplotype [BV209569/174 bp - DM47004.1/118 bp - DM46892/173 bp - DM46513/327 bp - D19S112/184 bp - D19S543/257 bp - APOC2/347 bp - DM45209/216 bp - DM45178/227 bp - DM45050/282 bp] is linked to the mutant/expanded allele. Consistent with the TP-PCR results, the paternal mutant haplotype was observed in embryos 2 and 3. On the other hand, embryo 1 showed a diplotype that was identical to the maternal diplotype, indicating that the sample was entirely maternal in origin. The results therefore indicate that none of the simulated embryos were suitable for transfer.

Overall, the *DMPK* TP-PCR genotypes were consistent with the dodecaplex marker PCR diplotypes, and the diagnoses were in complete agreement with the UK NEQAS proficiency test feedback on these samples.

## Discussion

Intrinsic endocrine issues related to DM1 such as primary hypogonadism in males and low response to ovarian stimulation in females may lead to poor outcomes when a couple with a DM1-affected spouse attempts to conceive naturally (Orngreen et al., 2012; Srebnik et al., 2014; Fernandez et al., 2017). Preimplantation genetic testing for monogenic disorders (PGT-M) of *in vitro*-fertilized embryos followed by selective transfer to the mother’s uterus of unaffected embryos is a feasible solution to increase the chances of an unaffected pregnancy. Compared to prenatal diagnosis, PGT-M avoids the dilemma and anguish that couples with an affected pregnancy experience, particularly when the disease severity and age of onset of DM1 cannot be predicted based solely on repeat size.

Standard repeat-spanning or flanking PCR to detect the normal *DMPK* allele of the affected parent is commonly employed in PGT-M for DM1 due to reliability issues in detecting the expanded allele, especially when performed directly on the limited genetic material of single cells (Sermon et al., 1997, 1998; Dean et al., 2001; Piyamongkol et al., 2001; Kakourou et al., 2007, 2008). This method was however useful only for couples with informative normal alleles, where the affected individual’s normal allele size differs from the unaffected partner’s normal alleles. Even when a couple’s normal alleles are informative, when the affected parent’s normal allele is not observed in an embryo, and there are insufficient informative markers to produce unambiguous haplotypes, the embryo cannot be transferred because it is not possible to determine if the embryo is unaffected or affected. TP-PCR has emerged as a better alternative as it detects the presence of an expanded allele regardless of its size, and hence is not reliant on the informativity of the normal allele. This feature is particularly advantageous considering that ~25% of the normal population are homozygous for a given normal allele (Brook et al., 1992; Gennarelli et al., 1998) and *DMPK* alleles of 5, 11, 12, and 13 repeats account for the vast majority of normal alleles (Brook et al., 1992; Davies et al., 1992; Goldman et al., 1994; Sizhong et al., 2000; Basu et al., 2001; Acton et al., 2007).

For couples whose normal alleles are uninformative, unidirectional TP-PCR has been performed to detect the expanded allele (Sermon et al., 2001; Kakourou et al., 2008, 2010). In 3–5% of DM1 cases, CCG, CTC, and GGC interruptions are present at either the 5′ or 3′ end of the CTG repeat tract, and this can potentially lead to false negative calls if only unidirectional TP-PCR is performed (Musova et al., 2009; Braida et al., 2010; Radvansky et al., 2011; Addis et al., 2012; Santoro et al., 2013, 2017). Our bidirectional (5′ and 3′) TP-PCR provides flexibility for use of both TP-PCR assays or either 5′ or 3′ assay to suit the specific needs of each couple.

When only direct mutation detection is employed, ADO of either of the DM1-affected parent’s alleles in an embryo will lead to inconclusive results, reduced numbers of embryos available for transfer, and consequently low pregnancy success rates. A high incidence of inconclusive results is particularly undesirable for DM1-affected women, given their poorer response to ovarian stimulation and consequently fewer embryos available for testing (Srebnik et al., 2014; Fernandez et al., 2017). Given that WGA using the multiple displacement amplification (MDA) method has an average ADO rate of 25% (Harton et al., 2011), there is a ~56% [(1 − ADO rate)^2^] probability that both parental *DMPK* alleles in a heterozygous embryo will be amplified (Renwick et al., 2007; Harton et al., 2011). Therefore, there is a 22% probability of ADO of the affected parent’s allele for every embryo. In our study, the ADO rate at the *DMPK* CTG repeat after WGA was observed to be 22.4%, which is in line with expectations (Harton et al., 2011) and suggests that single-cell MDA products are suitable templates for direct mutation detection in PGT-M of DM1. Transitioning from single blastomeres to multi-cell trophectoderm biopsy samples for PGT-M will further reduce the incidence of ADO. In a simulated PGT-M case from a previous UK NEQAS proficiency test where multiple lymphocytes were used to simulate a trophectoderm biopsy, ADO was not observed.

The ADO problem in DM1 PGT-M can also be satisfactorily addressed by employing a linked multi-marker haplotype analysis strategy to identify the normal and expanded *DMPK* alleles of the affected parent that are inherited by an embryo. Thus far, only four microsatellite markers have been described for use in PGT-M of DM1, one of which (APOC2, located approximately 0.8 Mb downstream of the CTG repeat) has been reported to have mutation-marker recombination (Kakourou et al., 2007). During genetic recombination between homologous chromosomes during meiosis, such that crossing over occurs between a marker and a gene mutation, linkage between the marker’s and gene’s alleles is exchanged. In the context of indirect diagnosis by linkage analysis, such a genetic recombination can lead to a misdiagnosis. The closer the physical distance between a polymorphic marker and a gene mutation, the less the likelihood of marker-mutation crossover/recombination occurring. This highlights the importance of using markers located as close as possible to the mutation site for reliable linkage analysis. Although we initially tested nine markers that were more proximal to the *DMPK* CTG repeat than APOC2, seven displayed low polymorphism information content (PIC), poor amplification results, or a complex peak pattern, and were therefore excluded from the multiplex marker panel (Lian et al., 2017). The remaining two markers, DM45958 and D19S543, located approximately 0.3 and 0.35 Mb downstream of the CTG repeat, respectively, are part of the dodecaplex marker panel. As the recombination possibility of these two markers is reduced to not more than 0.35%, they will be able to detect a recombination between APOC2 and the CTG repeat. Incidentally, no recombination was observed in the simulated PGT-M case between any of the 12 markers and the CTG repeat. Nevertheless, should the need arise, the seven excluded markers more proximal to the CTG repeat than APOC2 could still be useful to specific couples where they are tested to be informative.

When we performed a previously reported modified TP-PCR assay for DM1 (Kakourou et al., 2010), which involves simultaneous 5′ TP-PCR and standard PCR of the *DMPK* CTG locus and PCR of a linked microsatellite marker, D19S112, on two DM1 samples GM04567 and GM3989, we observed several challenges. Firstly, it was difficult to accurately size the normal alleles due to masking from the continuous TP-PCR peak pattern of the expanded allele. In comparison, both our 5′ and 3′ TP-PCR results showed unambiguous sizing of the normal alleles of both samples. Secondly, the 5′ TP-PCR of the previously published assay did not produce continuous peaks that extended beyond 50 repeats from the expanded alleles. In comparison, both our 5′ and 3′ TP-PCR assays generated continuous peaks from the expanded alleles of both samples that extended well beyond 50 repeats. Thirdly, the previously published assay employs only one linked marker which will frequently lead to situations where the marker is uninformative in a couple. In comparison, our multi-marker panel consists of 12 highly polymorphic markers (Lian et al., 2017), ensuring applicability to most, if not all, prospective couples. Fourthly, there was a significant difference in the yield of TP-PCR, standard PCR, and D19S112 products from the previously published single-tube reaction. The relatively poorer amplification of D19S112 required further enlargement of the electropherograms before the NED-labeled PCR products became visible. However, at such a high level of magnification, peaks caused by bleed-through from other fluorophore dye channels as a result of stronger amplification were also magnified, which could be mistaken for a second allele of the marker. This can result in incorrect genotyping and linkage of marker allele to mutation during allele phasing, leading to diagnostic ambiguity. Lastly, the modified TP-PCR method employs only a 5′ TP-PCR assay, which can potentially lead to false negative calls in cases where non-CTG interruptions are present at the 5′ end of the CTG repeat tract.

The TP-PCR and multi-marker assays provided complementary results for the simulated PGT-M case to unambiguously detect “embryos” positive for the paternal expanded *DMPK* allele. The multi-marker panel was also instrumental in resolving ambiguous/inconclusive results following TP-PCR, identifying an “embryo” as being entirely maternal in origin due to the presence of two maternal alleles for every marker and complete absence of paternal alleles.

One potential issue with marker DM46892 is that very large alleles of ≥172 bp amplify poorly, especially in heterozygous situations where the smaller allele’s size difference is ≥66 bp or ≥ 33 CA repeats. Furthermore, the fluorescent peak of such a large allele will be overlapped and overshadowed by fluorescent stutter peaks of marker BV209569, which is tagged with the same fluorophore. This was observed in the simulated PGT-M case, which resulted in the observation of ADO of the large 173-bp allele of DM46892. However, due to the high heterozygosity and PIC values observed for DM46892 (Lian et al., 2017), it was retained in the panel. In a situation where only one allele is observed but a second larger allele of ≥172 bp is suspected, as in the simulated PGT-M case, simplex PCR of this marker will be performed following the dodecaplex PCR to confirm the suspicion. Nevertheless, even with ADO at DM46892 or any other marker, haplotypes can still be confidently determined due to the high marker redundancy of the dodecaplex panel.

With five fully informative microsatellite markers available in the simulated PGT-M case, a diagnostic power of 98.4% {1 − [1 − (1 − *a*)^2^]^N^, *N* being the number of fully informative markers} is achieved (Renwick et al., 2007), assuming an ADO rate (*a*) of 25%. Using the maximum marker ADO rate of 26.8% observed in this study, the marker panel still yields a diagnostic power of 97.8%. The additional data from the five semi-informative markers also contributed to achieve a conclusive diagnosis in all “embryos” being tested. The number of panel markers actually used in PGT-M multiplex-PCR analysis can also be reduced to exclude the uninformative markers without having to modify reaction conditions.

The high number of markers in the panel increases the likelihood of its utility in any couple without having to customize case-specific marker panels, which can be costly and time-consuming. The high heterozygosity of each marker also increases the likelihood of finding multiple fully informative markers in each couple, which in turn increases its diagnostic power. Furthermore, the flexibility of the dodecaplex marker assay to be performed directly on single cells (Lian et al., 2017) makes it attractive as a rapid assay for linkage-based DM1 PGT-M.

In conclusion, our proposed DM1 PGT-M strategy increases diagnostic confidence through concordance analysis, minimizes misdiagnosis caused by ADO, exogenous DNA contamination, or genetic recombination, ultimately minimizing discard of potentially unaffected embryos caused by inconclusive results. This robust strategy can be applied without modification to most, if not all, at-risk couples.

## Author Contributions

ML has contributed to acquisition, analysis, and interpretation of data and drafting of the manuscript. CL has contributed to conception and design of the study and final approval of the version to be published. SC has contributed to conception and design of the study, analysis and interpretation of data, revising it critically for important intellectual content, and final approval of the version to be published.

### Conflict of Interest Statement

The authors declare that the research was conducted in the absence of any commercial or financial relationships that could be construed as a potential conflict of interest.
